# Acute Toxicity by Oral Co-Exposure to Palytoxin and Okadaic Acid in Mice

**DOI:** 10.3390/md20120735

**Published:** 2022-11-24

**Authors:** Silvio Sosa, Marco Pelin, Cristina Ponti, Michela Carlin, Aurelia Tubaro

**Affiliations:** Department of Life Sciences, University of Trieste, 34127 Trieste, Italy

**Keywords:** okadaic acid, palytoxin, oral co-exposure, acute toxicity, mice

## Abstract

The frequent occurrence of marine dinoflagellates producing palytoxin (PLTX) or okadaic acid (OA) raises concern for the possible co-presence of these toxins in seafood, leading to additive or synergistic adverse effects in consumers. Thus, the acute oral toxicity of PLTX and OA association was evaluated in mice: groups of eight female CD-1 mice were administered by gavage with combined doses of PLTX (30, 90 or 270 μg/kg) and OA (370 μg/kg), or with each individual toxin, recording signs up to 24 h (five mice) and 14 days (three mice). Lethal effects occurred only after PLTX (90 or 270 μg/kg) exposure, alone or combined with OA, also during the 14-day recovery. PLTX induced scratching, piloerection, abdominal swelling, muscle spasms, paralysis and dyspnea, which increased in frequency or duration when co-administered with OA. The latter induced only diarrhea. At 24 h, PLTX (90 or 270 μg/kg) and OA caused wall redness in the small intestine or pale fluid accumulation in its lumen, respectively. These effects co-occurred in mice co-exposed to PLTX (90 or 270 μg/kg) and OA, and were associated with slight ulcers and inflammation at forestomach. PLTX (270 μg/kg alone or 90 μg/kg associated with OA) also decreased the liver/body weight ratio, reducing hepatocyte glycogen (270 μg/kg, alone or combined with OA). No alterations were recorded in surviving mice after 14 days. Overall, the study suggests additive effects of PLTX and OA that should be considered for their risk assessment as seafood contaminants.

## 1. Introduction

Marine dinoflagellates include different harmful species producing a variety of non-proteinaceous toxins that can affect human health [1]. A serious sanitary problem is associated with consumption of shellfish and/or other edible marine organisms accumulating these toxins, which can cause diverse adverse effects linked to the chemical structure of the ingested compounds. An additional concern is raised by the simultaneous exposure to multiple algal toxins that can co-occur as marine foodstuff contaminants. In particular, the combined action of structurally different toxins could lead to additive or synergistic toxic effects due to their distinct mechanisms of action and/or to an increased absorption of some toxins consequent to intestinal damage induced by other harmful compounds [2,3]. Thus, as recommended by the European Food Safety Authority (EFSA), the hazard from combined exposure to multiple algal toxins should be characterized to update the risk assessment of these compounds as seafood contaminants [4].

In the Mediterranean Sea, a frequent algal toxin accumulated in edible bivalves is okadaic acid (OA) [5,6,7,8,9,10,11], a lipophilic polyether produced mainly by *Dinophysis* dinoflagellates causing a human gastrointestinal syndrome named diarrhetic shellfish poisoning (DSP) [12]. OA is a potent inhibitor of protein phosphatases acting on phosphoserine or phosphothreonine residues in cell proteins [13,14]. Inhibition of these enzymes by OA leads to a hyperphosphorylated level of different proteins, which in turn influences a series of cellular mechanisms, including those at the basis of diarrhea induction or tumor promotion [15]. In particular, diarrhea as an acute effect of OA is ascribed mainly to an increased phosphorylation level of proteins involved in ions secretion and of cytoskeletal/junctional elements of intestinal cells controlling paracellular permeability to solutes. This effect is assumed to cause a passive loss of fluids from the intestinal wall into the lumen, leading to diarrhea [8,16,17,18,19], but other mechanisms have been also suggested [18,20].

The hazard from oral exposure to OA has been characterized by toxicity studies in rodents. After single oral administration to mice, its median lethal dose (LD_50_) has been initially recorded between 1000 and 2000 μg/kg [21], and more recently at 880 and 760 μg/kg [22,23]. The toxin has been shown to induce tissue alterations visible by light or electron microscopy mainly at the gastrointestinal and hepatic levels, but also in the lymphoid tissues, kidneys, lungs, heart and brain [21,22,23,24,25,26,27,28,29,30,31,32,33]. Moreover, daily repeated oral OA administration to mice (185 or 1000 μg/kg, for 7 days) has been shown to induce alterations in the forestomach and, at the highest dose also in the liver, pancreas and lymphoid organs, with ultrastructural changes in cardiac muscle cells [34,35].

In the last decades, palytoxin (PLTX) and its analogues (ovatoxins) have been frequently detected along the Mediterranean coasts during *Ostreopsis* blooms, mainly *Ostreopsis* cf. *ovata* [36,37,38,39,40,41,42,43,44]. In this geographical area, *Ostreopsis* blooms and the relevant toxins have been often associated with respiratory, cutaneous and ocular adverse effects in humans after inhalation of marine aerosol and/or direct contact to seawater [45,46,47,48], but no cases of seafood poisoning ascribed to PLTXs have been documented thus far. On the contrary, severe and even lethal foodborne poisonings, characterized by gastrointestinal, neuromuscular, cardiac and respiratory symptoms, have been ascribed to PLTXs in tropical and subtropical areas [48,49,50,51,52,53].

PLTX is a highly toxic compound acting on Na^+^/K^+^-ATPase, an electrogenic transmembrane pump of animal cells involved in the maintenance of cellular osmotic equilibrium and membrane potential, crucial for cell volume regulation and signal transduction. The toxin converts the pump into a non-selective cation channel leading to passive transmembrane cations flux, which in turn triggers direct or indirect cytotoxic effects [54,55,56,57]. The acute oral toxicity of PLTX has been demonstrated by in vivo studies in mice showing LD_50_ values ranging from 510 to 767 μg/kg [58,59,60], with tissue alterations visible by light microscopy in the forestomach, liver and pancreas, and ultrastructural changes in myocardium, skeletal muscle and kidneys [59,60]. After daily oral administration to mice for 7 days, PLTX induced lethal effects at doses ≥30 μg/kg, with tissue alterations at gastrointestinal, hepatic, pulmonary, cardiac and the splenic level [61]. Moreover, prolonging PLTX administration to 28 days, an LD_50_ of 0.44 µg/kg and a no-observed-adverse-effect level (NOAEL) of 0.03 µg/kg have been determined in mice, recording macroscopic and microscopic alterations in the gastrointestinal tract at doses ≥0.1 μg/kg [62].

To protect shellfish consumers, the European food legislation provides the maximum admitted limits in seafood for different marine biotoxins, including OA and its analogues, of which the maximum permissible level has been set at 160 μg OA equivalents/kg shellfish meat [63]. On the contrary, PLTXs are still not regulated. Only the European Food Safety Authority elaborated a scientific opinion and derived an oral acute reference dose (ARfD) of 0.2 µg/kg body weight for the sum of PLTX and its analogue ostreocin-d, recommending a threshold value of 30 µg/kg shellfish meat [64]. Nevertheless, PLTXs have been detected in Mediterranean shellfish or other edible marine organisms also at levels exceeding the recommended threshold of 30 µg/kg [65,66,67,68,69,70], which may result in human exposure to PLTXs amounts exceeding the ARfD derived by EFSA. An additional concern for human health is the possible co-occurrence of PLTX and OA in seafood, due to the combined adverse effects of these toxins differing in chemical structure and mechanism of action. Thus, the hazard from oral co-exposure to PLTX and OA should be characterized to improve their risk assessment as seafood contaminants. In this study, the acute oral toxicity by co-exposure to PLTX and OA was evaluated in mice: the effects of three PLTX doses (30, 90 or 270 μg/kg) combined with a dose of OA inducing mild diarrhea (370 μg/kg) have been compared to those of the same doses of each individual toxin.

## 2. Results

The study was carried out in two experimental phases (experiment 1 and 2). Experiment 1 was a pilot study, in which mice were administered with the 30 μg/kg PLTX (low dose) combined with 370 μg/kg OA, or with the same doses of each individual toxin. In experiment 2, the doses of PLTX were increased at 90 μg/kg (mid dose) and 270 μg/kg (high dose), while the dose of OA remained unchanged (370 μg/kg). Since experiments 1 and 2 were parts of a single study, the obtained results are cumulatively described for a comprehensive overview.

### 2.1. Mortality

Within 24 h after administration, no lethality was recorded in mice treated with OA (370 μg/kg) or with the low PLTX dose (30 μg/kg) as single toxins, whereas the mid- or the high dose of PLTX alone (90 and 270 μg/kg) induced the death of 2/8 mice in each group. Similarly, after the toxin co-administration, only the mid- or the high PLTX dose combined with OA resulted in lethal effects (2/8 mice in each group) (Table 1).

During the 14-day recovery period, lethal events occurred in mice administered with the mid-dose of PLTX as a single toxin (1/3 mice, day 8) and in those co-administered with the high PLTX dose and OA (1/3 mice, day 5) (Table 1).

### 2.2. Signs of Toxicity

Administration of OA (370 μg/kg) induced only transitory diarrhea, visible within 2 h from its administration (4/16 mice). Within 24 h, no signs of toxicity were noted in mice administered with the low PLTX dose (30 μg/kg). Increasing the dose at 90 μg/kg, PLTX induced recurrent episodes of scratching in one mouse, which recovered within a few hours, whereas 2/8 mice that died within 24 h showed piloerection, sedation and muscular spasms, associated with abdominal dilation in one mouse. These signs were accompanied by tremors, jumping, paralysis (mainly of the hind limbs) and dyspnea in 1–3/8 mice administered with the high dose of PLTX (270 μg/kg) as an individual toxin. Combined administration of PLTX and OA induced signs of toxicity also in mice receiving the low PLTX dose (transitory diarrhea: 2/8 mice; recurrent episodes of scratching within 2 h: 3/8 mice; piloerection: 1/8 mice). These signs of toxicity, associated with more severe ones, such as tremors, loss of righting reflex, paralysis dyspnea and muscular spasms, occurred in mice administered with the mid- or the high PLTX dose combined with OA, some of them being longer as compared to those recorded in mice administered only with PLTX. In particular, the mid-PLTX dose and OA induced transitory diarrhea (3/8 mice), piloerection and abdominal dilation (1/8 mice), as well as sedation, muscular spasms, loss of righting reflex, dyspnea and/or paralysis (mainly of the hind limbs) in 2/8 mice that died. These signs, accompanied by tremors, also occurred in spontaneously dead mice and in some mice sacrificed 24 h after the co-administration of the high PLTX dose and OA (1–4/8 mice) (Table 2).

During the recovery period, signs of toxicity were recorded only in two spontaneously dead mice: progressive sedation and dilated abdomen occurred in 1/3 mice treated with the mid- PLTX dose and in 1/3 mice co-administered with the high PLTX dose combined with OA (data not shown).

### 2.3. Body Weight

**Figure 1:**
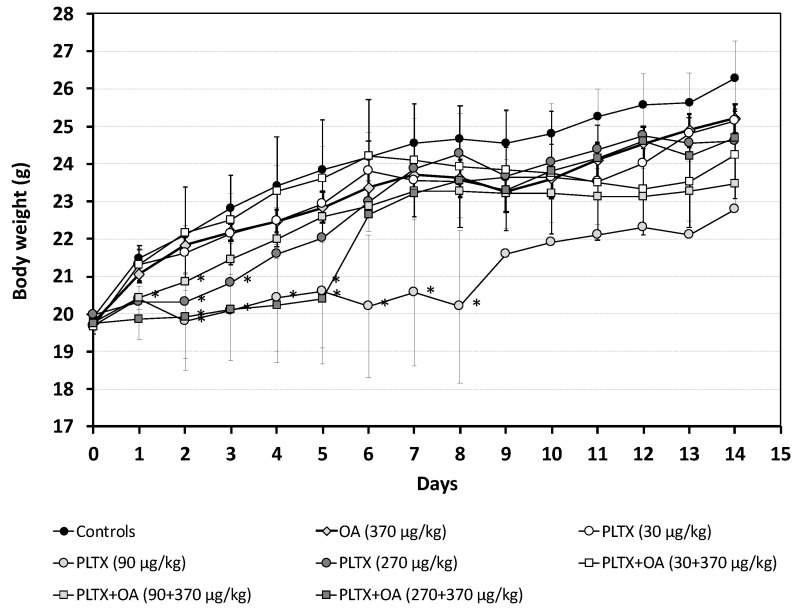
Body weight of mice from the day of treatment (D 0) up to 14 days of recovery (D 14). At days 0 and 1: data are the mean ± S.E. of 16 values (controls, OA 370 μg/kg) or 8 values (other groups of treatment, with exception at day 1: data are the mean ± S.E. of 6 values for PLTX 90 μg/kg, PLTX 270 μg/kg, PLTX+OA 90+370 μg/kg and PLTX+OA 270+370 μg/kg); from days 2 to 14: data are the mean ± S.E. of 3 values (with exception at day 8 and 5: data are the mean of 2 values for PLTX 90 μg/kg and for PLTX+OA 270+370 μg/kg, respectively); * *p* < 0.05 at the analysis of variance, in comparison with controls.

As compared to controls, no significant body weight changes were recorded in mice within 24 h from the administration of OA (370 μg/kg) or the low PLTX dose (30 μg/kg) as single toxins. On the other hand, a slight but significant body weight reduction (about 5%) was recorded in mice exposed to the mid- or the high PLTX dose (90 or 270 μg/kg), both as a single toxin or combined with OA (Figure 1).

During the 14-day recovery, no significant changes in body weight were recorded between controls and mice administered with OA or the low PLTX dose as single toxins, whereas a significant body weight reduction occurred in those administered with the mid- PLTX dose (10–18% up to day 8, when one mouse died) or with the high PLTX dose (8–9%, up to day 3). The combined toxins administration resulted in a significant body weight reduction only in mice co-exposed to OA and mid-PLTX dose (8%, at day 3) or to OA and the high PLTX dose (10–15% up to day 5, when one mouse died) (Figure 1).

### 2.4. Food Consumption

**Figure 2:**
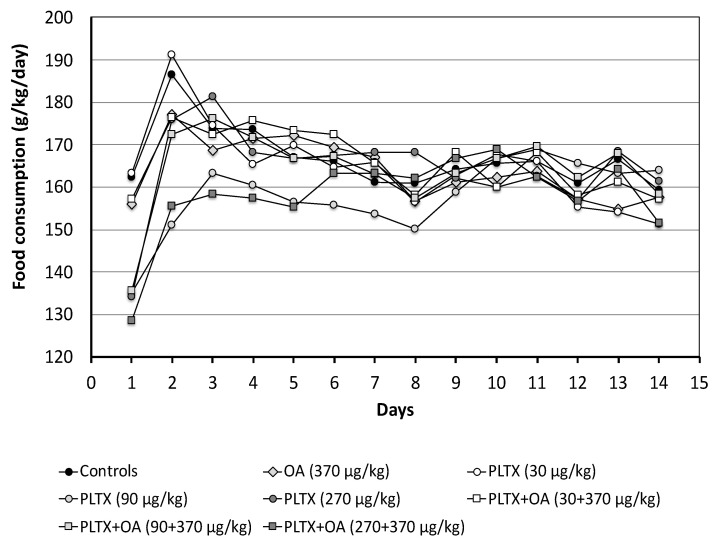
Food consumption by mice recorded one day after treatment (day 1) and during the recovery period up to day 14. Data represent the daily food consumption by each group of mice (g/kg body weight/day).

Food consumption was calculated from the ratio between the diet eaten by the group of mice and the total body weight. At 24 h from administration, mice treated with the mid- or the high PLTX dose (90 or 270 μg/kg) showed a reduced food intake (17% in each group), as compared to controls. Similarly, mice co-administered with these PLTX doses combined with OA showed reduced food consumption (16% and 21%, respectively) (Figure 2).

A reduced food intake was also recorded during the 14-day recovery in mice treated with the mid-PLTX dose as a single toxin (9–19% reduction, up to day 8) and in those co-administered with OA and the high PLTX dose (7–17% reduction, up to day 5). In the latter, food consumption up to day 5 was lower than that of mice administered with the same dose of PLTX or OA as individual toxins (7–13% or 10–12%, respectively) (Figure 2).

### 2.5. Gross Pathology and Relative Organs Weight

At 24 h, no macroscopic signs were recorded in the main organs of mice treated with the low PLTX dose (30 μg/kg) as an individual toxin, whereas exposure to 90 or 270 μg/kg PLTX induced redness of the intestinal wall (1/5 mice at each dose). Administration of OA as an individual toxin (370 μg/kg) induced only a pale fluid accumulation in the proximal tract of the small intestinal lumen (6/10 mice). This finding was also recorded in 4/5 mice co-administered with OA and low PLTX dose (30 μg/kg), accompanied by intestinal wall redness (3/5 mice). These macroscopic changes co-occurred in all the mice administered with the mid- or high PLTX dose combined with OA, with digestive tract redness extended to the gastric wall. The frequency of intestinal and gastric wall redness was significantly higher in mice co-exposed to the mid- or high PLTX dose and OA, as compared to that recorded in mice treated with the corresponding doses of PLTX as a single toxin (Table 3).

During the 14-day recovery, macroscopic alterations (gastrointestinal wall redness, pale fluid accumulation in the small intestinal lumen and swollen abdominal cavity) were noted only in spontaneously dead mice (1/3 mice treated with 90 μg/kg PLTX alone, day 8; 1/3 mice treated with OA combined with 270 μg/kg PLTX, day 5). No macroscopic alterations were noted in surviving mice sacrificed at the end of the withdrawal period (data not shown).

**Figure 3:**
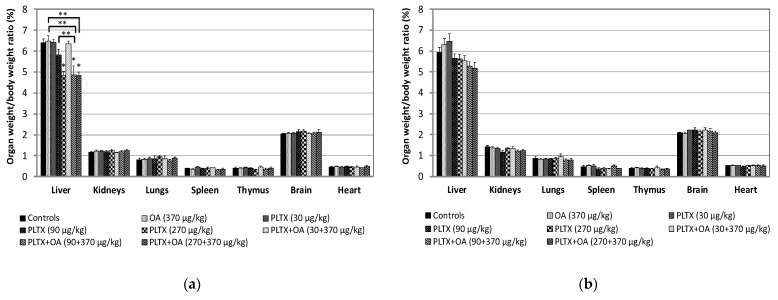
Organ to body weight ratios of mice recorded within 24 h after treatment (**a**) and during the recovery period up to 14 days from the treatment (**b**). (**a**) Data are the mean ± S.E. of 10 mice (controls, OA 370 μg/kg) or 5 mice (other groups of treatment); (**b**) Data are the mean ± S.E. of 6 mice (controls, OA 370 μg/kg) or 3 mice (other groups of treatment); * *p* < 0.05 at the analysis of variance, in comparison with controls; ** *p* < 0.05 at the analysis of variance, as compared between mice administered with PLTX (90 μg/kg) or OA (370 μg/kg) as single toxins and those co-administered with PLTX and OA (90 and 370 μg/kg), or between mice treated with OA (370 μg/kg) as a single toxin and those co-administered with PLTX and OA (270 and 370 μg/kg).

Concomitantly to necropsy, the relative weight of the main organs (organ weight/body weight ratio) was determined. As compared to controls, at 24 h from administration, only a reduced liver/body weight ratio was recorded in mice given the high PLTX dose as an individual toxin (24%) and in those co-administered with OA and the mid- or the high PLTX dose (25% in each group). The relative liver weight of mice co-administered with OA and the mid-PLTX dose was also significantly lower than that of mice treated with the same doses of each individual toxin (25% or 17%, respectively). Moreover, the relative liver weight of mice co-administered with OA and the high PLTX dose was significantly lower (25%) than that of mice administered with only OA (Figure 3a).

No significant changes in relative organ weight were recorded at the end of the recovery period (Figure 3b).

### 2.6. Blood Chemistry

The blood volume sampled from the majority of mice administered with the low PLTX dose, alone or associated with OA, was not sufficient for the blood chemistry analyses, and the relevant data are not reported.

**Figure 4:**
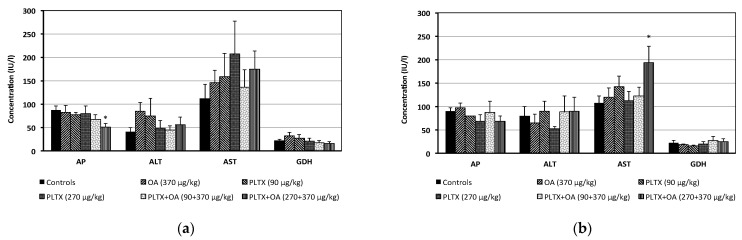
Serum levels of alkaline phosphatase (AP), alanine aminotransferase (ALT), aspartate aminotransferase (AST) and glutamate dehydrogenase (GDH) recorded in mice within 24 h after treatment (**a**) and during the recovery period up to 14 days from treatment (**b**). (**a**) Data are the mean ± S.E. of 8 mice (controls, OA 370 μg/kg) or 5 mice (other groups). (**b**) Data are the mean ± S.E. of 6 mice (controls, OA 370 μg/kg) or 3 mice (other groups); * *p* < 0.05 at the analysis of variance, in comparison with controls.

Within 24 h from administration, no significant changes in the blood chemistry parameters occurred in mice administered with each toxin alone, as compared to controls. Only a not significant increase in AST serum level was recorded in mice administered with OA or the mid- and the high PLTX dose as individual toxins (30%, 41% or 84%, respectively). A not significant increase in AST was also recorded in mice co-administered with OA and the mid- or the high PLTX dose (21% and 55%, respectively). In addition, mice given the high PLTX dose combined with OA showed a significant reduction (42%) of AP serum level (Figure 4a).

After 14-day recovery, only a significant increase in AST serum level (81%) was recorded in mice co-administered with OA and the high PLTX dose, as compared to controls. The AST serum concentration in these mice was also higher than that of mice administered with the corresponding dose of OA or PLTX as individual toxins (62% or 72%, respectively), even at marginal significance level (0.05 < *p* < 0.10) (Figure 4b).

### 2.7. Light Microscopy

**Figure 5:**
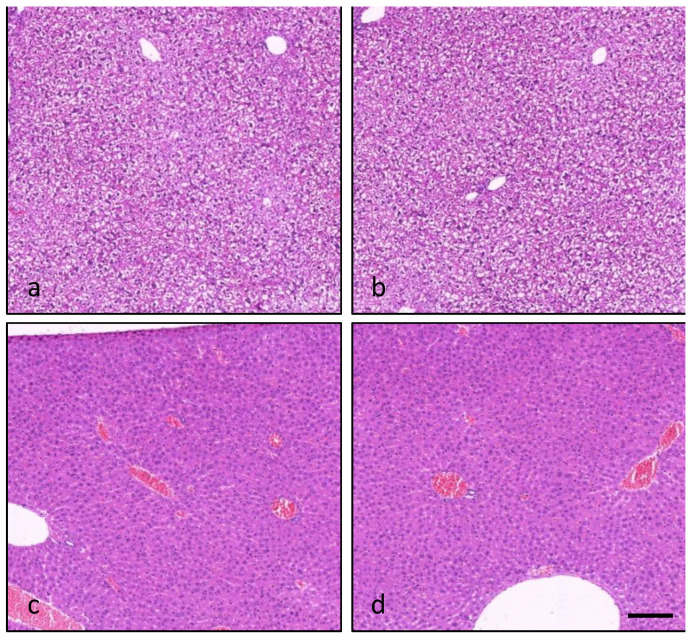
Light micrographs of the liver from a control mouse (**a**), a mouse administered with OA (370 μg/kg; (**b**) or PLTX (270 μg/kg; (**c**)) and a mouse co-administered with OA and PLTX (370 and 270 μg/kg, respectively; (**d**)), showing fine vacuolated and granulated hepatocytes’ cytoplasm (**a**,**b**) and its reduction (**c**,**d**). Images are representative of 5 mice. Hematoxylin–eosin stain; magnification 10×; bar: 100 μm.

Histological analysis showed tissue changes in the liver and/or forestomach of mice sacrificed 24 h after the administration of PLTX alone or combined with OA. At hepatic level, the fine vacuolated and granulated cytoplasm observed in hepatocytes of controls was reduced in mice (5/5) administered with the high PLTX dose (270 μg/kg), singly or combined with OA. This finding is compatible with a reduced glycogen content provoked by PLTX administration (Figure 5).

**Figure 6:**
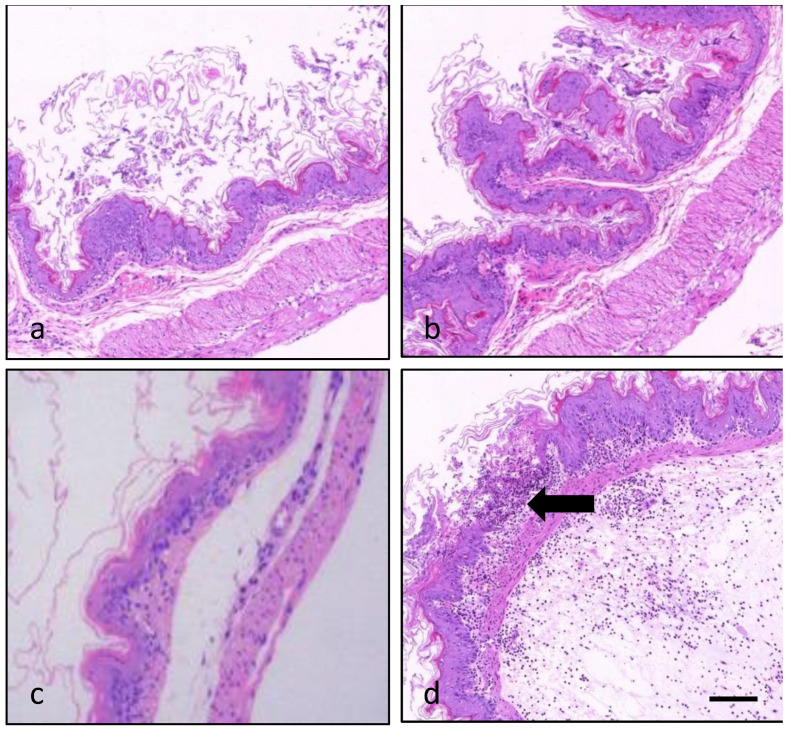
Light micrographs of the forestomach from a control mouse (**a**), a mouse administered with OA (370 μg/kg; (**b**)) or PLTX (270 μg/kg; (**c**)) and a mouse co-administered with OA and PLTX (370 and 270 μg/kg, respectively; (**d**)), showing a slight ulcer ((**d**), arrow) accompanied by inflammatory cells infiltration and edema in the submucosa ((**d**), bottom right part). Images are representative of 5 mice (**a**,**b**) and 2 mice (**c**,**d**). Hematoxylin–eosin stain; magnification 10×; bar: 200 μm.

At gastric level, some mice co-exposed to the mid- (90 μg/kg) or the high PLTX dose (270 μg/kg) and OA showed slight to mild ulcers in the non-glandular part, accompanied by inflammatory cell infiltration (2/5 mice, in each group). An inflammatory reaction typical of gastritis with edema and polymorphonuclear cells infiltrate was particularly evident in the forestomach of 2/5 mice co-administered with OA and the high PLTX dose (Figure 6).

No tissues changes were recorded in mice sacrificed after 14-day recovery (data not shown).

## 3. Discussion

In the last decades, harmful algal blooms have increased throughout the world’s seawaters with negative impacts to human health and local economies. In the Mediterranean Sea, blooms of *Dinophysis* dinoflagellates producing OA and/or its analogues frequently occurred since the nineteen-eighties with frequent episodes of DSP [5,6,7,8,9,10,11]. In the last decades, blooms of *Ostreopsis* (mainly *Ostreopsis* cf. *ovata*) were also recorded along the Mediterranean coasts [39,40,41,42,43,44]. Concomitantly to these events, PLTX and its analogues had been detected in different edible marine organisms, also at concentrations exceeding the maximum admitted level proposed by EFSA [64,65,66,67,68,69,70]. In these situations, OA and PLTX could co-occur in seafood, raising concerns for possible additive or synergistic effects in consumers. Thus, to characterize the hazard by oral co-exposure to OA and PLTX, a comparative acute oral toxicity study was carried out in mice: the effects of OA (370 μg/kg) and PLTX (30, 90 or 270 μg/kg) co-administration were compared to those of each individual toxin.

Within 24 h, the mid- or the high dose of PLTX (90 or 270 μg/kg) induced lethal effects, even individually or combined with OA (370 μg/kg), with the same incidence (2/8 mice in each group). Lethality data indicate no additive or synergistic effects between PLTX and OA and put in evidence the high lethal potency of PLTX. Its individual lethal dose (90 μg/kg) was lower than 300 μg/kg, previously recorded as NOEL (no observed effect level) in mice under the same experimental conditions [59], as well as those lower than 100 μg/kg, previously recorded as a non-lethal dose for mice, even prolonging the time of observation to 96 h [60]. This finding highlights the variable inter-individual sensitivity to PLTX, already noted in mice as variable lethality after repeated oral administration [61], and suggested by epidemiological data in humans [48,50,51,52,53]. The different inter-individual sensitivity to PLTX could be related, at least in part, to genetic factors, such as a variable inter-individual expression of Na^+^/K^+^-ATPase subunits’ isoforms, as suggested by an in vitro toxicogenetic study on human monocytes [71].

As compared to controls, administration of the mid- or the high PLTX dose resulted in a reduced food intake (17% in each group), comparable to that of mice exposed to the same PLTX doses combined with OA (16% and 21% reduction, respectively). These data cannot be statistically analyzed, being derived from food consumption by each whole group of animals and not from the mean consumption by each mouse, but they support the adverse effects of PLTX when combined with OA. In parallel, administration of these PLTX doses resulted in a slight but significant body weight loss (about 5%), which was not influenced by OA co-administration. In addition, at these doses, PLTX caused a series of clinical signs (scratching, piloerection, sedation, muscular spasms, tremors, jumping, paralysis of the hind limbs and/or dyspnea) suggesting the involvement of the neuromuscular system, some of them previously noted after single oral administration of PLTX or 42-hydroxy-PLTX in mice [58,59,60,72]. On the other hand, OA induced only visible transitory diarrhea (4/16 mice), comparable in incidence with that recorded in mice co-exposed to OA and each dose of PLTX (2–3/8 mice). Mice co-administered with the two toxins showed the combined signs of toxicity induced by each individual toxin. Although they appeared longer and/or more frequent as compared to those recorded after the individual toxin administration, the significance of these differences is not statistically evident due to the limited number of mice. Two signs (scratching and piloerection) occurred even in mice receiving the low PLTX dose (30 μg/kg) combined with OA, but were not noted after the administration of the same doses of each individual toxin. Thus, globally, these findings suggest an additive effect between PLTX and OA, supported also by necropsy and histology findings.

Necropsy showed distinct macroscopic changes in the gastrointestinal tract of mice administered with OA or the mid- and high PLTX dose: pale fluid accumulation in the proximal tract of the small intestine occurred in mice exposed to OA, whereas redness of the small intestinal wall was noted in mice administered with PLTX. These alterations co-occurred in mice co-exposed to OA and even to the low PLTX dose, indicating an additive effect. Moreover, mice receiving OA with the mid- or the high PLTX dose showed redness of the digestive tract extended to the gastric wall, where histological analysis revealed the presence of forestomach lesions (slight focal ulcers and inflammatory cells infiltrate; 2/5 mice in each group). An inflammatory reaction of the forestomach submucosa typical of gastritis, with edema and polymorphonuclear cells infiltrate, was more evident in mice co-exposed to the high PLTX dose and OA. Gastric lesions (weak erosions and slight fluid accumulation) were previously recorded in mice orally administered with 200 μg/kg PLTX or its analogue ostreocin-d [73], while acute forestomach inflammation was noted after higher doses (≥424 μg/kg) of PLTX or 42-hydroxy-PLTX oral administration [59,72]. Gastric alterations were previously recorded in mice after single oral OA administration. An acute inflammation of the forestomach squamous mucosa and erosions of the fundic mucosa were recorded after the administration of 2–10 μg OA/mouse [26], whereas light erosion of surface forestomach epithelial cells was noted at 150 μg/kg [30]. In addition, bloody content in the gastric lumen and/or wounds were noted after the administration of OA doses ≥750 μg/kg [23], whereas acute forestomach submucosal inflammation with epithelial vacuolar degeneration and/or reactive hyperplasia of keratinized epithelium were recorded at 1000 μg/kg [21]. Furthermore, our study indicates the stomach as a target of PLTX and OA association, with an additive effect between the two toxins. While the individual toxins did not induce any gastric alteration visible by light microscopy, the mid- or the high PLTX dose combined with OA induced ulceration and inflammation in the forestomach, probably due to the combined local irritant action. Even though an adverse effect at the forestomach is relevant for rodents rather than for humans, which are devoid of this anatomical structure, the combined irritant effects of PLTX and OA at the digestive tract should be considered to update the risk assessment of these toxins as seafood contaminants.

Administration of the high dose of PLTX as a single toxin or its co-administration even at the mid- or the high dose with OA also affected the liver, as shown by the decreased liver/body weight ratio. As compared to controls, the high PLTX dose induced 24% reduction of liver/body weight ratio, comparable to that recorded after the mid- or the high PLTX dose combined with OA (25% in each group). In addition, the liver/body weight ratio in mice co-exposed to the mid- PLTX dose and OA was significantly lower (17%) than that of mice exposed to the same PLTX dose as the individual toxin, indicating its additive effect with OA. On the other hand, histological analysis suggests a decreased glycogen content in hepatocytes of almost all the animals given the highest PLTX dose, both as a single toxin or combined with OA. Furthermore, these findings were not accompanied by increased serum levels of enzyme indices of liver injury. Only a reduction of alkaline phosphatase (42%) was recorded in mice co-exposed to OA and the high PLTX dose, but the enzyme levels remained within the physiological range of mice [74]. A decreased glycogen content in liver cells was previously noted in mice after single oral administration of PLTX doses ≥600 µg/kg [59] or OA doses ≥500 µg/kg [23]. OA had been previously shown to induce ultrastructural alterations in liver cells at doses ≥500 µg/kg [23], necrotic foci and lipid vacuoles at 700 µg/kg [33], and cytoplasmic vacuolation at 2000 µg/kg [21]. Although our findings do not show a hepatotoxic effect for OA, they confirm the liver as a target of PLTX at a dose as low as 270 µg/kg, which is lowered to 90 µg/kg if associated with OA.

Adverse effects due to PLTX administration, even with lethal outcomes, occurred during the 14-day recovery in mice administered with the mid-dose of PLTX or co-administered with the high PLTX dose and OA: 1/3 mice died at days 8 and 5, respectively. Signs of toxicity (progressive sedation and abdominal dilation) and macroscopic alterations (gastrointestinal wall redness and pale fluid accumulation in the small intestine) were noted only in these spontaneously dead mice. Other findings were recorded only in these groups of mice (reduced body weight and food consumption; 81% increase in AST serum level only in those co-administered with the high PLTX dose and OA). Furthermore, no liver or gastric alterations were observed in surviving mice at the end of the withdrawal period, suggesting a complete recovery of the toxic effects.

## 4. Materials and Methods

### 4.1. Toxins and Chemicals

Okadaic acid (purity grade: 98%) and palytoxin (purity grade: >90%) were purchased from Wako Chemical GmbH (Neuss, Germany). The toxins, dissolved in 95% aqueous ethanol, were diluted with phosphate-buffered saline (PBS), reducing ethanol concentration to 1.8% (*v*/*v*). If not otherwise indicated, analytical grade solvents and other chemicals were from Sigma Aldrich (Milan, Italy).

### 4.2. Animals and Experimental Conditions

Female CD-1 mice (18–20 g body weight, 4 weeks old) were purchased from Harlan Laboratories (S. Pietro al Natisone, Udine, Italy). Animals were acclimatized for one week before the experiments at controlled temperature (21 ± 1 °C) and humidity (60–70%), with a fixed artificial light cycle (7.00 a.m.–7.00 p.m.). Animals were caged using dust-free poplar chips for bedding and fed with the standard diet for rodents (Harlan Laboratories; S. Pietro al Natisone, Udine, Italy). The diet composition, as indicated by the supplier, was: proteins (18.5%), fats (5.5%), fibers (4.5%), hashes (6.0%), non-nitrogen compounds (53.5%) and water (12.0%). Water and feed were provided ad libitum during the entire duration of the experiments.

Experiments were carried out at the University of Trieste (Italy), in compliance with the Italian Decree no. 116/1992 as well as the EU Directive (2010/63/EU) and the European Convention ETS 123. The experimental study was approved by the University Body for Animal Well-being (OPBA) of the University of Trieste and the Italian Ministry of Health (decree no. 112/2013-B of 14 May 2013).

### 4.3. Dose Selection and Experimental Design

*Selection of the toxins’ doses*. The dose of OA (370 μg/kg) was selected on the basis of a preliminary study as a non-lethal dose inducing fluid accumulation in the small intestine and mild visible diarrhea. At this dose, OA co-administration with PLTX would avoid or reduce the excretion of the latter through feces, which could limit its effects. In our experimental conditions, the single oral administration of this OA dose to CD-1 female mice induced mild transitory diarrhea within few hours and/or fluid accumulation in the small intestine notable within 24 h by necropsy. PLTX was administered at three doses (30, 90 and 270 μg/kg), selected on the basis of previous findings after its acute and repeated oral toxin administration to CD-1 female mice. In particular, in our experimental conditions: (i) after acute oral PLTX administration, the NOEL (no observed effect level) was 300 μg/kg [59]; (ii) after daily oral administration for 7 days, the NOAEL (no observed adverse effect level) was 3 μg/kg/day, whereas toxic and even lethal effects occurred after four daily oral administrations at 30 μg/kg/day [61]. Hypothesizing an additive or synergistic effect, a pilot experiment (experiment 1) was carried out co-administering 370 μg/kg OA and 30 μg/kg PLTX. Then, in a subsequent study (experiment 2), PLTX was administered at 3-fold increased doses (90 and 270 μg/kg), the dose of OA remaining unchanged (370 μg/kg).

*Experimental design.* At day 0 (D0), groups of 8 mice were administered by gavage with a single dose of OA (370 µg/kg) combined with PLTX (30, 90 or 270 µg/kg), or with the corresponding doses of each toxin alone. Control mice were administered with the vehicle (10 ml/kg PBS, containing 1.8% ethanol, *v*/*v*). After administration, mice were monitored up to 24 h, when subgroups of 5 mice were sacrificed. Subgroups of 3 mice were maintained for a 14-day withdrawal period, recording clinical signs, body weight and food consumption, daily in the morning. Food consumption was calculated from the amount of diet eaten by all the mice of each group divided by the total body weight. At the scheduled times of sacrifice (24 h after the treatment: day 1, D1; 14 days after the treatment: day 14, D14), mice were anesthetized by intraperitoneal injection of tiletamine/zolazepam (20 mg/kg; Zoletil^®^; Virbac; Milan, Italy) and xylazine (5 mg/kg; Virbac; Milan, Italy), and bled to death through the abdominal aorta to collect blood for chemistry analyses (see Section 4.4). Then, mice were necropsied, and liver, heart, lungs, kidneys, spleen and brain were removed and weighed. Samples of these organs and other tissues (see Section 4.5) were fixed in neutral buffered formalin for the histological analysis. Similarly, animals that died spontaneously were immediately weighed, and the blood was collected for chemistry analyses; the main organs and tissues were weighed and/or fixed for the histological analysis. The groups of treatment, the number of mice and the scheduled sacrifice are reported in Table 4.

### 4.4. Blood Chemistry Analyses

Blood was allowed to clot for 15 min at room temperature and centrifuged at 2000× *g* for 10 min at 4 °C. Serum was separated and stored at −80 °C until the analyses were carried out. Using an automatized analyzer (AU400 Olympus with Beckman Coulter reagents), the following parameters were determined: alkaline phosphatase (AP), alanine aminotransferase (ALT), aspartate aminotransferase (AST), glutamate dehydrogenase (GDH), total cholesterol, creatinine, glucose, total proteins, triglycerides, urea, albumin, globulins, albumin/globulins ratio, sodium ions, potassium ions, chloride ions, calcium ions and inorganic phosphorus (Pi).

### 4.5. Histological Analysis

Heart, liver, lungs, kidneys, spleen, stomach, duodenum, jejunum, colon, rectum, pancreas, thymus, cerebrum, cerebellum, spinal cord, uterus, ovaries and skeletal muscle (soleus) were fixed in neutral buffered 10% formalin, embedded in paraffin and cut in sections of 5 µm. Sections were deparaffinized, rehydrated and stained with hematoxylin and eosin, following standard techniques for histological analyses by light microscopy. Pictures were obtained with a Nikon eclipse *i* 50 microscope equipped with a DS-Vi1 digital camera and NIS-Elements Microscope Imaging version 3.2 Software (Nikon Instruments; Tokyo, Japan).

### 4.6. Statistical Analysis

Data are expressed as mean ± standard error (S.E.). Significant differences between groups were calculated by one-way analysis of variance, followed by the Dunnett’s test for multiple comparisons of unpaired data, accepting *p* values lower than 0.05 as significant. Frequency of clinical signs and of macroscopic changes at gross pathology is expressed as number of mice showing the sign(s) or alteration(s)/number of treated mice, and significant differences between groups were calculated by Fisher’s exact test, accepting *p* values lower than 0.05 as significant.

## 5. Conclusions

The acute toxicity study in mice demonstrates that single oral co-administration of PLTX (30, 90 or 270 µg/kg) and OA (370 µg/kg) resulted in a LOAEL (lowest observed adverse effect level) of 30 µg/kg PLTX combined with 370 µg/kg OA. Moreover, it highlights a high oral lethality of PLTX, which is not influenced by OA co-administration. The other severe clinical signs of toxicity by PLTX and OA association are ascribed mainly to PLTX and are increased in frequency and/or duration by OA coadministration, which suggests an additive effect between these toxins. These aspects should be considered to update the risk assessment of PLTX and OA as seafood contaminants.

## Figures and Tables

**Table 1:** Lethality and survival times of mice after acute oral PLTX and/or OA administration.

PLTX Dose (μg/kg)	OA Dose (μg/kg)	Lethality at 24 h ^1^ (Survival Time, h:min)	Lethality during 14-Day Recovery ^1^ (D: Day of Death)
0	0	0/16 (--)	0/6 (--)
0	370	0/16 (--)	0/6 (--)
30	0	0/8 (--)	0/3 (--)
90	0	2/8 (04:52–06:50)	1/3 (D8)
270	0	2/8 (01:48–04:47)	0/3 (--)
30	370	0/8 (--)	0/3 (--)
90	370	2/8 (00:39–02:30)	0/3 (--)
270	370	2/8 (03:46–06:49)	1/3 (D5)

^1^ Number of dead animals/number of treated animals.

**Table 2:** Signs of mice within 24 h from PLTX and/or OA oral administration, their frequency and interval of occurrence after the toxin(s) administration.

Sign	Controls	OA 370 ^1^	PLTX 30 ^1^	PLTX 90 ^1^	PLTX 270 ^1^	PLTX+OA 30+370 ^1^	PLTX+OA 90+370 ^1^	PLTX+OA 270+370 ^1^
Diarrhea	0/16 (--)	4/16 (01:12–05:25)	0/8 (--)	0/8 (--)	0/8 (--)	2/8 (01:06–05:30)	3/8 (01:00–06:10)	3/8 (00:55–06:20)
Scratching	0/16 (--)	0/16 (--)	0/8 (--)	1/8 (00:30–02:06)	1/8 (00:24–02:18)	3/8 (00:30–02:18)	1/8 (0:24–02:.25)	1/8 (00:20–02:35)
Piloerection	0/16 (--)	0/16 (--)	0/8 (--)	2/8 (01:30–05.48)	2/8 (00.35–03:12)	1/8 (01:48–24:00)	2/8 (00:20–01:35)	3/8 (00:18–11:30)
Righting reflex loss	0/16 (--)	0/16 (--)	0/8 (--)	0/8 (--)	0/8 (--)	0/8 (--)	2/8 (00:25–01:35)	2/8 (00:30–05:15)
Sedation	0/16 (--)	0/16 (--)	0/8 (--)	2/8 (03:00–05:48)	2/8 (01:00–03:18)	0/8 (--)	2/8 (00:15–01:36)	2/8 (00:30–05:15)
Tremors	0/16 (--)	0/16 (--)	0/8 (--)	0/8 (--)	2/8 (01:30–03:12)	0/8 (--)	0/8 (--)	2/8 (01:18–05:15)
Jumping	0/16 (--)	0/16 (--)	0/8 (--)	0/8 (--)	2/8 (01:35–03:00)	0/8 (--)	0/8 (--)	0/8 (--)
Paralysis	0/16 (--)	0/16 (--)	0/8 (--)	0/8 (--)	3/8 (01:42–10:12)	0/8 (--)	2/8 (00:42–01:35)	4/8 (00:48–11:30)
Dyspnea	0/16 (--)	0/16 (--)	0/8 (--)	0/8 (--)	2/8 (01:42–03:12)	0/8 (--)	2/8 (01:12–01:36)	2/8 (01:18–05:15)
Muscular spasms	0/16 (--)	0/16 (--)	0/8 (--)	2/8 (04:48–05:48)	2/8 (01:30–03:12)	0/8 (--)	2/8 (00:24–01:35)	2/8 (00:24–05.12)
Abdomen dilation	0/16 (--)	0/16 (--)	0/8 (--)	1/8 (03:00–06:48)	1/8 (01:30–04:42)	0/8 (--)	1/8 (02:30–24:00)	2/8 (01:24–15:25)

^1^ Dose: μg/kg; data are expressed as number of animals showing the sign(s)/total number of animals; in brackets: mean interval of sign occurrence (h:min) after the toxin(s) administration.

**Table 3:** Macroscopic alterations in mice within 24 h from PLTX and/or OA oral administration and their frequency.

Alteration	Controls	OA 370 ^1^	PLTX 30 ^1^	PLTX 90 ^1^	PLTX 270 ^1^	PLTX+OA 30+370 ^1^	PLTX+OA 90+370 ^1^	PLTX+OA 270+370 ^1^
Intestinal redness	0/10	0/10	0/5	1/5	1/5	3/5 *	5/5 *^,§^	5/5 *^,§^
Intestinal fluid	0/10	6/10 *	0/5	0/5	0/5	4/5 *^,§^	5/5 *^,§^	5/5 *^,§^
Gastric redness	0/10	0/10	0/5	0/5	0/5	0/5	5/5 *^,§^	5/5 *^,§^

^1^ Dose: μg/kg; data are expressed as the number of animals showing the alteration(s)/total number of animals; * *p* < 0.05 at Fisher’s exact test, as compared to controls; ^§^ *p* < 0.05 at Fisher’s exact test, as compared to the corresponding dose of each toxin alone (or to PLTX alone, for intestinal fluid).

**Table 4:** Groups of treatment, number of treated mice and of scheduled sacrificed mice.

Group	PLTX Dose (μg/kg)	OA Dose (μg/kg)	N° of Treated Animals	N° of Sacrificed Animals (24 h)	N° of Sacrificed Animals (14 Days)
*Experiment 1*					
1	0	0	8	5	3
2	0	370	8	5	3
3	30	0	8	5	3
4	30	370	8	5	3
*Experiment 2*					
1	0	0	8	5	3
2	0	370	8	5	3
3	90	0	8	5	3
4	270	0	8	5	3
5	90	370	8	5	3
6	270	370	8	5	3

## Data Availability

Not applicable.
